# Supplementary material for: Laccase-Carrying Polylactic Acid Electrospun Fibers, Advantages and Limitations in Bio-Oxidation of Amines and Alcohols
Source: J Funct Biomater. 2022 Dec 31;14(1):25. doi: 10.3390/jfb14010025 (PMC9866953; doi:10.3390/jfb14010025)
Supplement: Supplementary file 1 [file jfb-14-00025-s001.zip › jfb-2089672-supplementary.pdf]

Laccase-Carrying Polylactic Acid Electrospun Fibers, Advantages and Limitations in Bio-Oxidation of Amines and Alcohols

Valentina Giraldi [1], Maria Letizia Focarete [1,2,*] and Daria Giacomini [1,2,*]

[1] Department of Chemistry "Giacomo Ciamician", University of Bologna, Via Selmi 2, 40126 Bologna, Italy
[2] Health Sciences & Technologies (HST) CIRI, University of Bologna, Via Tolara di Sopra 41/E, 40064 Ozzano dell'Emilia, Italy
* Correspondence: marialetizia.focarete@unibo.it (M.L.F.); daria.giacomini@unibo.it (D.G.)

Supplementary Material

Table of contents

1) Laccase activity assay
2) Bradford assay details
3) Optimization of the electrospinning method for the preparation of PLLA-LTv mats
4) SEM images of the obtained electrospun PLLA-LTv mats
5) ATR-FTIR analysis
6) Water contact angle (WCA) measurements
7) Thermogravimetric analysis (TGA)

1) Laccase activity assay

Laccase *Tv* activity was determined spectrophotometrically, following the oxidation of catechol to benzoquinone (Scheme S1).

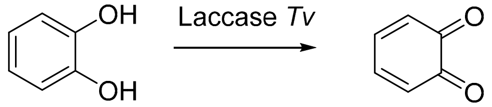

Scheme S1. *Catechol oxidation to benzoquinone catalyzed by Laccase Tv.*

The activity tests were conducted in a 96 polystyrene well plate.
For free laccase activity a modified literature procedure [1] was used: in each well, 100 μL of a 20 mM catechol solution in acetate buffer (0.5 M, pH=4.5), 90 μL of acetate buffer and 10 μL of a 1 mg/mL laccase *Tv* solution in acetate buffer were added, in triplicate, giving a total volume of 200 μL. Blank solution was made of 100 μL of catechol solution and 100 μL of acetate buffer. Immediately after laccase solution addiction, the plate was shaken for 1 min and 30 s and absorbance was measured at Λ=405 nm in 20 seconds intervals for 2 hours, using the stepping and kinetic mode of the instrument (Thermo Scientific Multiskan EX). Data were collected by Ascent software and analyzed by Excel.

Quantitative determination of the free enzyme activity was obtained by the formula:

$$Activity\ (\mu mol \cdot min^{-1} \cdot mg^{-1}) = \frac{{}^{dA}/_{dt}\,(min^{-1})}{\varepsilon\ (mL \cdot \mu mol^{-1} \cdot cm^{-1}) \cdot l\ (cm) \cdot [E](mg \cdot mL^{-1})}$$

where dA/dt is the slope of Abs-Time curve in the initial phase of the reaction (approximately 0-10 min), where the trend is linear. ε is the molar extinction coefficient of benzoquinone at λ=405 nm (1,26 $mL \cdot \mu mol^{-1} \cdot cm^{-1}$), [E] is the enzyme concentration inside the plate well and *l* is the optical path length of the radiation through the sample, in this case *l*=0.5 cm.

For PLLA-LTv activity measurements, samples of approximately 1x1 cm were weighted and soaked in 2 mL of acetate buffer (0.5 M, pH= 4.5), and then placed upon an orbital shaker at 400 rpm for 15 minutes. The mats were then added to 2 mL of 5 mM catechol solution in acetate buffer (0.5 M, pH=4.5). Aliquots of 200 μL were taken from the reaction mixture and placed in a 96 well plate for absorbance measure at λ=405 nm, in duplicate. Immediately after the measure, the aliquots were put back into the reaction mixture, to keep the same reaction volume [2]. The first measure was done 10 minutes after the beginning of the reaction, and then every 5 minutes.
Several formulas were used to evaluate the activity of immobilized laccase and the immobilization efficiency [2]:

1) Activity of PLLA-LTv, expressed in U per mg of mat:

$$Activity\ \left(\frac{U(\mu mol \cdot min^{-1})}{mg_{mat}}\right) = \frac{k \cdot V_{tot}(L) \cdot 10^6}{M_{mat}(mg) \cdot \varepsilon\left(\frac{L}{mol \cdot cm}\right) \cdot l\ (cm)}$$

Where: k is the slope of the Abs-time curve in the initial phase of reaction (10-30 min), $V_{tot,}$ is the total volume of reaction medium expressed in L, M_{mat} is the mass of the piece of mat used for the activity test expressed in mg, ε is the extinction coefficient of benzoquinone at λ=405 nm (1260 M^{-1} cm^{-1}), 10^6 is the conversion factor to transform mol to μmol and l is the optical path length of the radiation expressed in cm.

2) Specific activity of immobilized laccase, expressed in U per mg of loaded protein and obtained dividing the activity of PLLA-LTv by the mg of loaded laccase:

$$Specific\ activity\ \%\ \left(\frac{U}{mg}\right) = \frac{Activity\ of\ PLLA - LTv\left(\frac{U}{mg_{mat}}\right)}{Loaded\ protein\ \left(\frac{mg}{mg_{mat}}\right)} \cdot 100$$

3) Retained Activity % as the portion of free laccase activity that is retained after the immobilization process and expressed as:

$$Retained\ activity\ \% = \frac{Specific\ activity\ of\ immobilized\ laccase\ \left(\frac{U}{mg}\right)}{Specific\ activity\ of\ free\ laccase\ \left(\frac{U}{mg}\right)} \cdot 100$$

2) Bradford assay details

Bradford assay is based on the reaction between aromatic residues of a protein with Comassie Blue dye leading to a blue coloured complex (λmax = 595 nm), that was detected spectrophotometrically on Thermo Scientific Multiskan EX instrument fixed length at 620 nm) [3]. The volume ratio between the sample and the Bradford reagent influences the linear range and the limit of detection (LOD): we used microvolumes and a microassay procedure in which a 1:1 ratio is used, to detect 0-100 μg/mL concentrations, being this range more suitable to our problem.

The assay requires a calibration curve that is usually made with standard solutions of BSA, being BSA relatively inexpensive and easy to obtain. Instead, we did some preliminary tests with a series of Laccase *Tv* standard solutions in both water and acetate buffer 0.5 M., to understand if a linear response is possible with the microassay procedure.

There was a linear response in the 10-75 μg/mL range using Laccase *Tv* standards in water. On the other hand, there was no linearity in the case of acetate buffer 0.5 M, being Bradford reagent sensible to this concentration of buffer. Lowering the buffer concentration to 0.1 M allowed us to have a good linearity. However, it must be noted that, in both cases, the method was characterized by a poor sensitivity that could be due to laccase itself and its aromatic residues content.

For the enzyme leaching studies, external standards method was used, both in water and acetate buffer 0.1 M. Calibration curves were obtained using four standards solutions, with six measured absorbance values for every standard, taking into account both the error on

the dispensed volume (since every solution was dispensed in triplicate) and the error on the standard solution preparation (since standards were made in duplicate) (Figure S1).

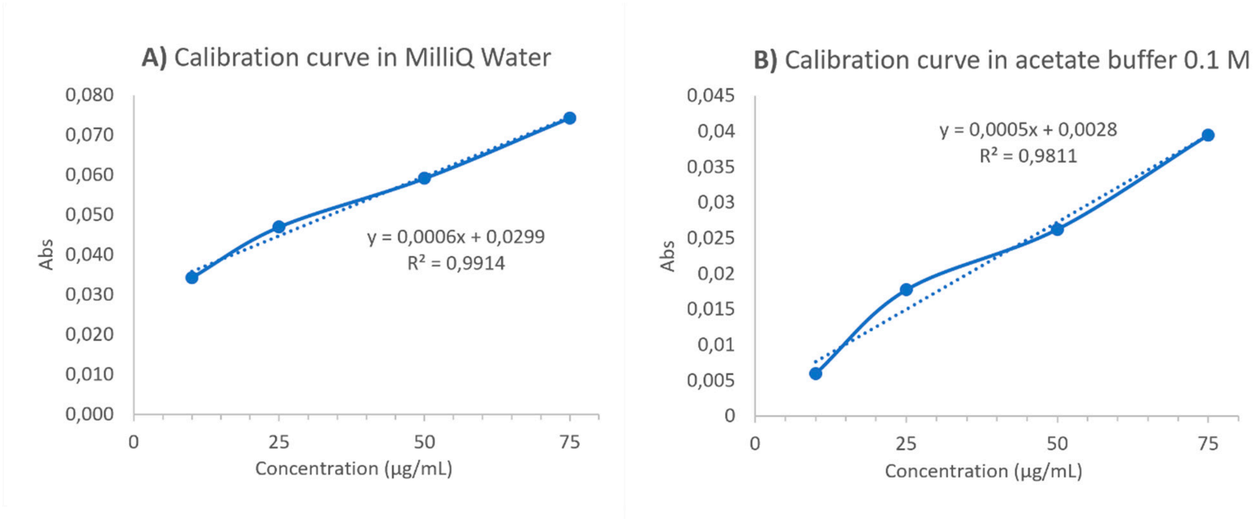

Figure S1. Examples of calibration curves in water (A) and acetate buffer 0.1 M (B).

In this assay, a calibration curve is necessary every time the samples are measured; thus, the standard solutions were always dispensed together with the samples. The limit of detection (LOD) for every calibration curve was obtained with the following formula:

$$LOD = 3{,}3 \cdot \frac{Standard\ error\ of\ the\ regression}{Coefficent\ of\ the\ x\ variable}$$

Where the standard error and the coefficient for x were obtained by the linear regression statistics adopting a 95% interval of confidence.
Depending on the specific linear regression that was made, LOD may differ from one test to another but generally it is the 5-15 µg/mL range.

Different samples of PLLA-LTv were used. Concentration of laccase into the release medium was determined by interpolation of the absorbance value of the sample into the linear regression and expressed in µg/mL. Then, the released µg of laccase was normalized by the weight of the mat sample. When possible, values are expressed as the average with its standard deviation.

3) Optimization of the electrospinning method for the preparation of PLLA-LTv mats

Starting from our knowledge of PLLA electrospinning, a 20% $^{w}/_{v}$ PLLA solution in DCM was prepared. Pluronic©127 was added in a 10% $^{w}/_{w}$ ratio with respect to PLLA. As a first attempt to form an emulsion, 4,8 % $^{v}/_{v}$ of MilliQ water was added to the organic phase (Entry 1). Vortexing at 2000 rpm for some minutes led to a stable emulsion with a homogeneous

appearance that was charged on a glass syringe and electrospun. Electrospinning parameters were varied until beads-free fibers were observed at the optical microscope. This was achieved with a 17 kV applied voltage, a flow rate of 23 μL/min and a tip-collector distance of 20 cm, in a vertical configuration with an aluminium foil as the collector. However, agglomerates tended to form at the needle tip during the electrospinning, probably due to the high viscosity of the solution and the high volatility of DCM. In entry 2, the viscosity and the volatility of the solution were lowered by dissolving PLLA in 13% $^{w}/_{v}$ concentration in a 9:1 mixture of DCM and DMF, with the same quantity of Pluronic©F127 and MilliQ water. In this case, we were not able to observe beads-free fibers even after changing the process parameters. In entry 3, raising the concentration of PLLA to 15% $^{w}/_{v}$ whilst maintaining constant the others solution parameters allowed us to obtain beads-free fibers without an excessive formation of agglomerates at the needle tip. Optimized electrospinning parameters were the same used for entry 1.

In all these tests, the quantity of water in the emulsion was set to 4.8 % $^{v}/_{v}$. Being this a very low quantity of water, it could be difficult to solubilize much laccase into it efficiently. In entry 4, 20% $^{v}/_{v}$ of water was added to understand the upper limit on water amount. In this case, it was not possible to obtain a stable emulsion. An intermediate water content (9% $^{v}/_{v}$) was adopted successfully for the electrospinning of PLLA-LTv mats.

Table S1: Optimization of solution and electrospinning parameters.

**Entry**	**PLLA (% w/v)**	**Pluronic©F127 (% w/w)**	**Organic phase Solvent mixture**	**MilliQ H_2O (% v/v)**	**Notes**
**1**	20	10	DCM	4.8	High viscosity of the solution
**2**	13	10	DCM: DMF 9:1	4.8	Presence of beads in the fibers
**3**	15	10	DCM: DMF 9:1	4.8	Optimal parametrers
**4**	15	10	DCM: DMF 9:1	20	Not able to form a stable emulsion

4) SEM images of the obtained electrospun PLLA-LTv mats

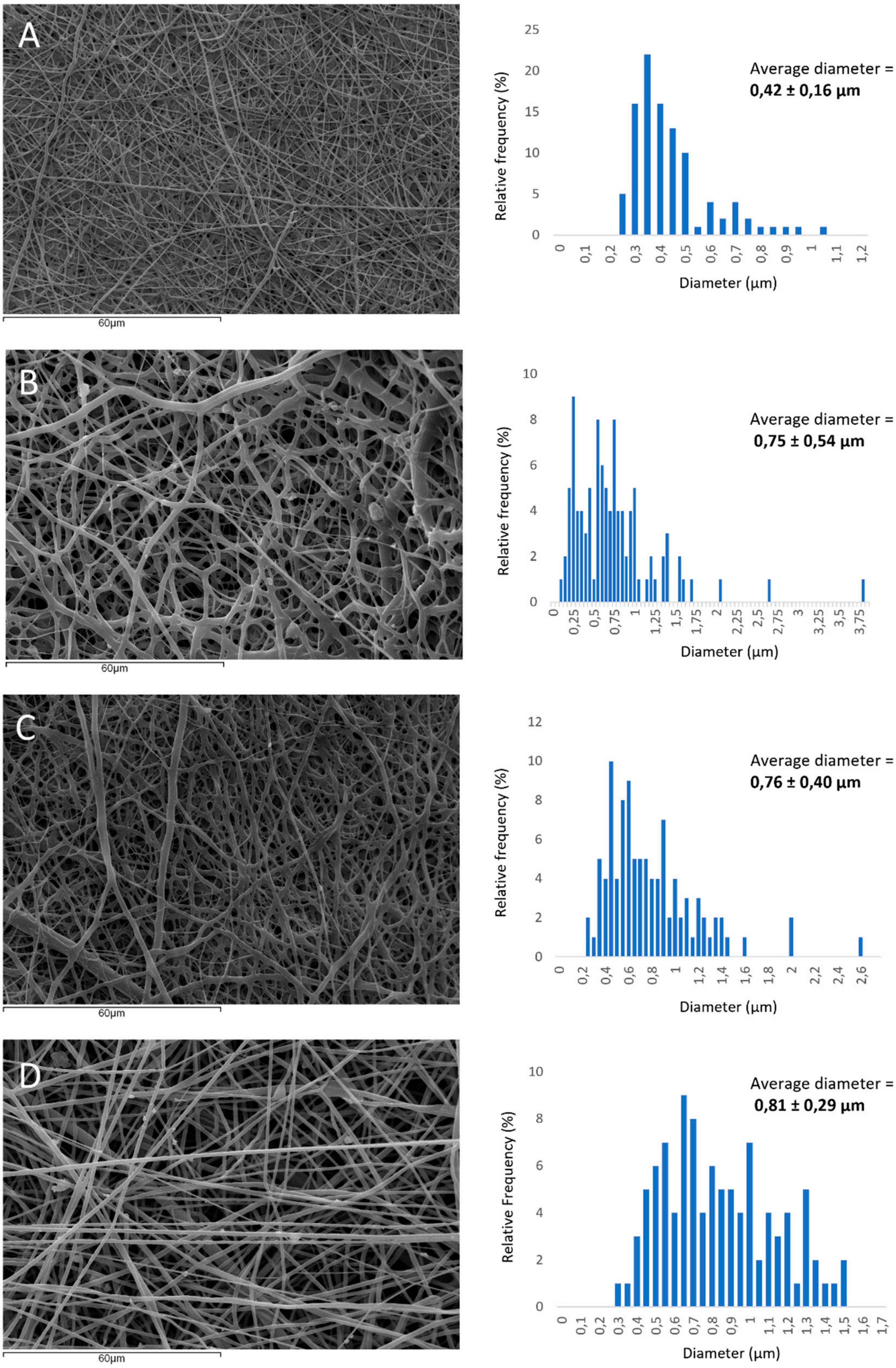

Figure S2. On the left, SEM images at 1000x magnification of PLLA-Pluronic©F127 (A), PLLA-LTv-A (B), PLLA-LTv-B (C) and PLLA-LTv-C (D); on the right, their diameter distribution. Average diameter is expressed as the average value with its standard deviation.

5) ATR-FTIR analysis

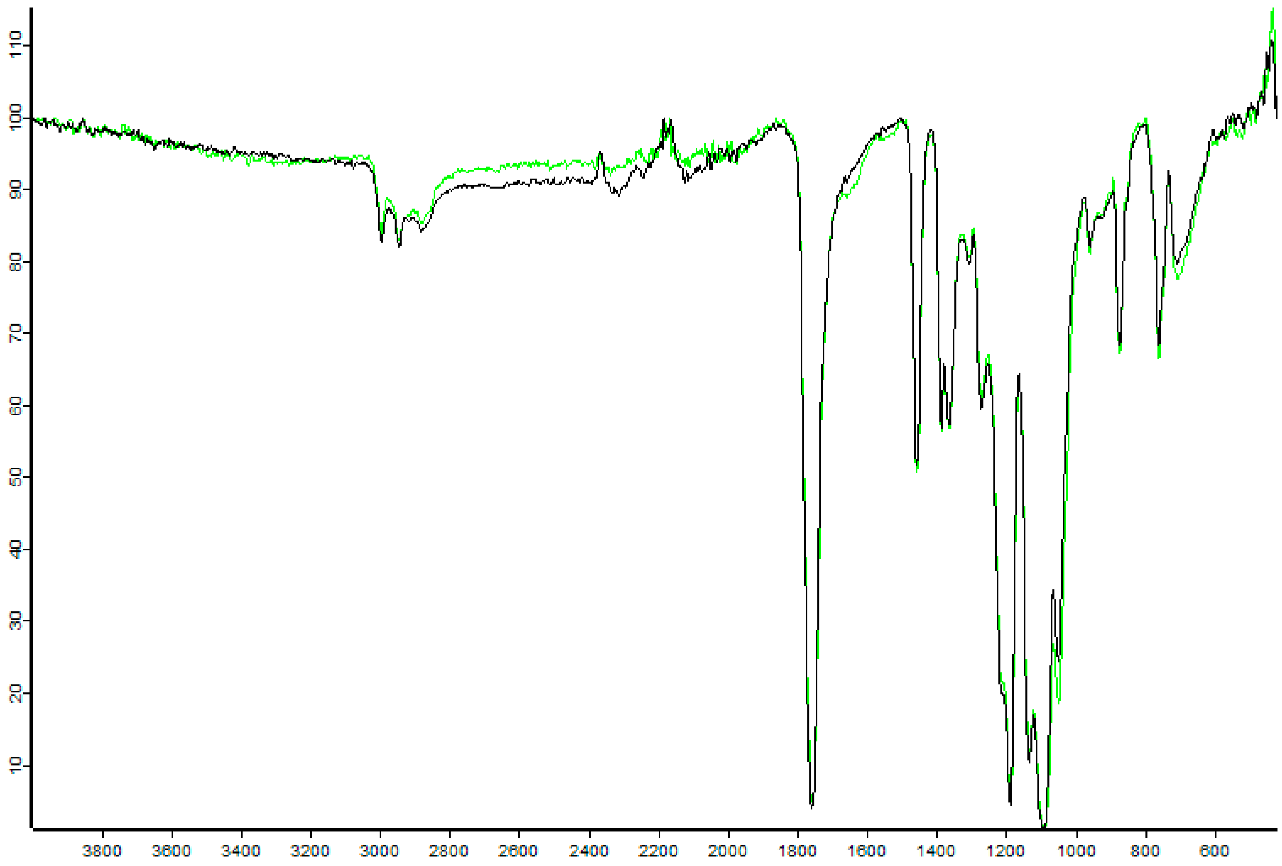

Figure S3. Overlapping of ATR-FTIR spectra of PLLA-Pluronic©F127 (black) and PLLA-LTv-C (green).

6) Water contact angle (WCA) measurements

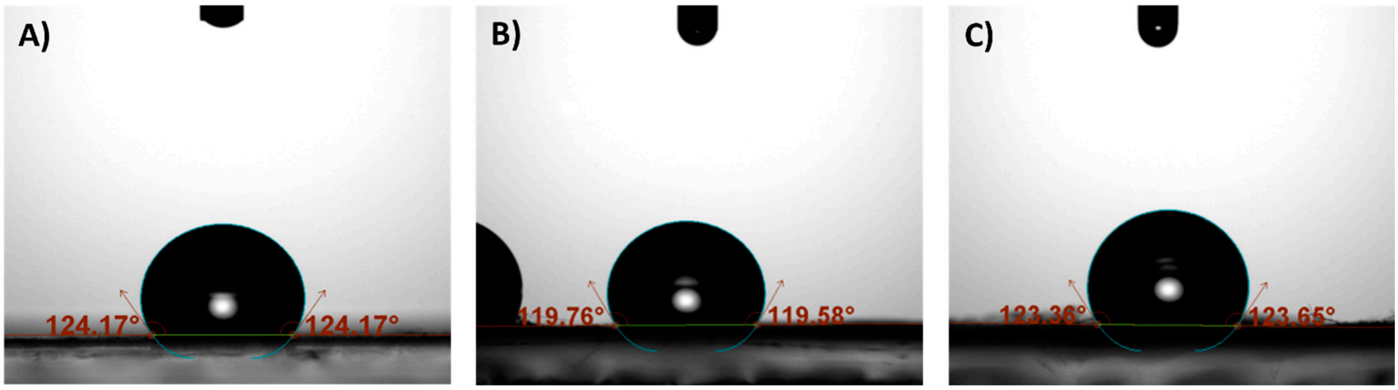

Figure S4. Pictures of representative water contact angle measurements on A) Plain PLLA B) PLLA-Pluronic®F127 and C) PLLA-LTv-B mats.

Table S2. Values of water contact angle determined for each mat. Results are given as the average value with its standard deviation.

**Mat**	**Water contact angle (°)**<br>**(Average ± Standard Deviation)**
Plain PLLA	124,57 ± 6,94
PLLA-Pluronic®F127	119,67 ± 2,40
PLLA-LTv-B	122,65 ± 6,12

7) Thermogravimetric analysis (TGA)

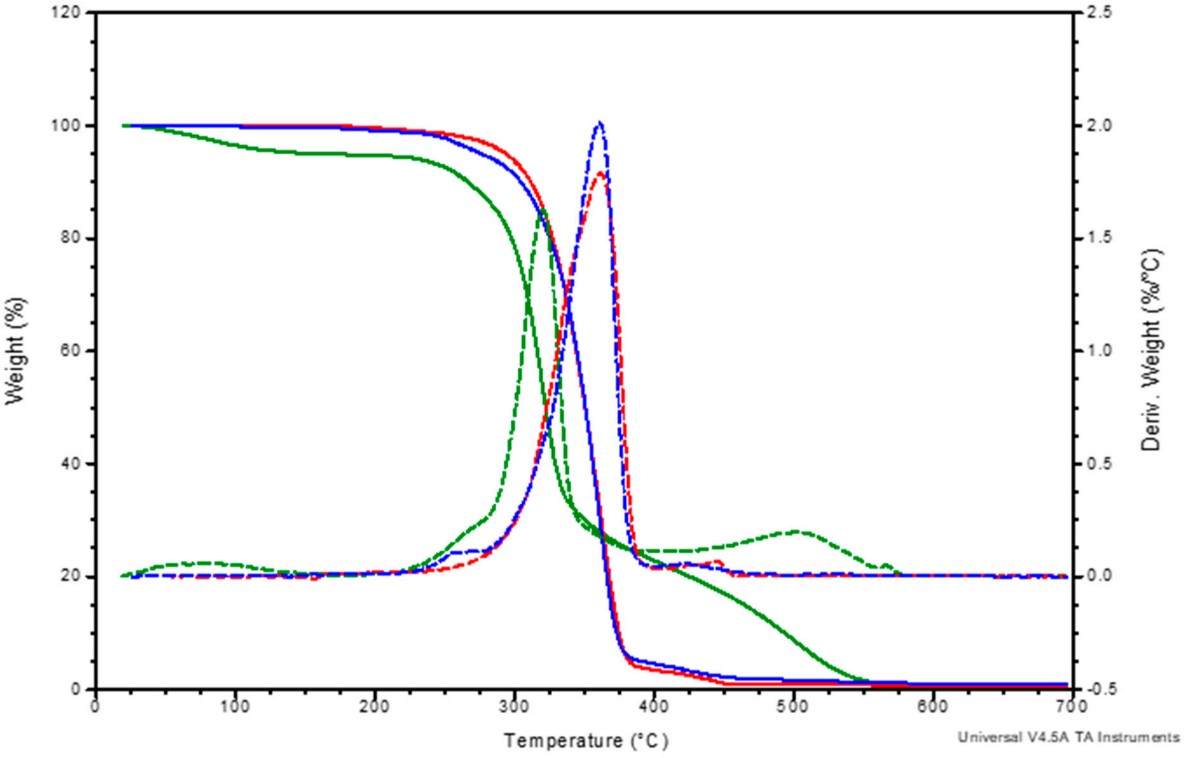

Figure S5. Overlay of the thermogravimetric curves of Laccase *LTv* (green), PLLA-Pluronic®F127 (red) and PLLA-LTv-C (blue) in air. Solid line: Weight %; dashed line: Derivative weight (%/°C).

References

[1] Matijošyté, I.; Arends, I. W.C.E.; de Vries, S.; Sheldon, R. A. Preparation and use of cross-linked enzyme aggregates (CLEAs) of laccases. *J. Mol. Catal. B: Enzym*. **2010**, *62*, 142-148 https://doi.org/10.1016/j.molcatb.2009.09.019

[2] Chen, L.; Zou, M.; Hong, F.F. Evaluation of Fungal Laccase Immobilized on Natural Nanostructured Bacterial Cellulose. *Front Microbiol.* **2015,** *6*, 1245 https://doi.org/10.3389/fmicb.2015.01245

[3] Bradford, M. A rapid and sensitive method for the quantitation of microgram quantities of protein utilizing the principle of protein-dye binding. *Anal. Biochem.* **1976**, *72*, 248–255. https://doi.org/10.1016/0003-2697(76)90527-3
